# Peanut Seed Coat Acts as a Physical and Biochemical Barrier against *Aspergillus flavus* Infection

**DOI:** 10.3390/jof7121000

**Published:** 2021-11-23

**Authors:** Leslie Commey, Theophilus K. Tengey, Christopher J. Cobos, Lavanya Dampanaboina, Kamalpreet K. Dhillon, Manish K. Pandey, Hari Kishan Sudini, Hamidou Falalou, Rajeev K. Varshney, Mark D. Burow, Venugopal Mendu

**Affiliations:** 1 Department of Plant and Soil Science, Fiber and Biopolymer Research Institute (FBRI), Texas Tech University, Lubbock, TX 79409, USA; Leslie.Commey@ttu.edu (L.C.); tktengey@yahoo.com (T.K.T.); chriscobos11@gmail.com (C.J.C.); Kamalpreet.Dhillon@ttu.edu (K.K.D.); 2 CSIR-Savanna Agricultural Research Institute (SARI), Nyankpala P.O. Box 52, Ghana; 3 Department of Plant and Soil Science, Texas Tech University, Lubbock, TX 79409, USA; Lavanya.Dampanaboina@ttu.edu (L.D.); md.burow@ttu.edu (M.D.B.); 4 International Crops Research Institute for the Semi-Arid Tropics, Hyderabad 502324, India; M.Pandey@cgiar.org (M.K.P.); H.Sudini@cgiar.org (H.K.S.); R.K.Varshney@CGIAR.ORG (R.K.V.); 5 International Crops Research Institute for the Semi-Arid Tropics, Niamey B.P. 873, Niger; H.Falalou@cgiar.org; 6 State Agricultural Biotechnology Centre, Centre for Crop and Food Innovation, Murdoch University, Murdoch, WA 6150, Australia; 7 Texas A&M AgriLife, Lubbock, TX 79401, USA; 8 Department of Plant Sciences and Plant Pathology, Montana State University, Bozeman, MT 59717, USA

**Keywords:** seed coat, peanut, aflatoxin, *Aspergillus flavus*

## Abstract

Aflatoxin contamination is a global menace that adversely affects food crops and human health. Peanut seed coat is the outer layer protecting the cotyledon both at pre- and post-harvest stages from biotic and abiotic stresses. The aim of the present study is to investigate the role of seed coat against *A. flavus* infection. In-vitro seed colonization (IVSC) with and without seed coat showed that the seed coat acts as a physical barrier, and the developmental series of peanut seed coat showed the formation of a robust multilayered protective seed coat. Radial growth bioassay revealed that both insoluble and soluble seed coat extracts from 55-437 line (resistant) showed higher *A. flavus* inhibition compared to TMV-2 line (susceptible). Further analysis of seed coat biochemicals showed that hydroxycinnamic and hydroxybenzoic acid derivatives are the predominant phenolic compounds, and addition of these compounds to the media inhibited *A. flavus* growth. Gene expression analysis showed that genes involved in lignin monomer, proanthocyanidin, and flavonoid biosynthesis are highly abundant in 55-437 compared to TMV-2 seed coats. Overall, the present study showed that the seed coat acts as a physical and biochemical barrier against *A. flavus* infection and its potential use in mitigating the aflatoxin contamination.

## 1. Introduction

Mycotoxins are produced by *Aspergillus*, *Fusarium*, and Penicillium species as a defense response as well as in response to environmental changes [1]. Aflatoxins (B_1_, B_2_, G_1_, G_2_) are produced by *Aspergillus flavus* and *A. parasiticus*, and have been considered as the most potent mycotoxins, especially aflatoxin B_1_ (AFB_1_). AFB_1_ has been recognized as a class I human carcinogen by the International Agency for Research on Cancer [2]. Aflatoxin poses significant health problems in humans, such as liver cancer, growth impairment in children, and acute aflatoxicosis in developing countries [3]. In developed countries, the average dietary exposure to aflatoxin is generally below 1 ng/kg body weight per day, while those of sub-Saharan nations in Africa are above 100 ng/kg body weight per day [4]. In addition to the health risk, it has been reported that 25% or more of global food crop production per annum is not used for food consumption due to aflatoxin contamination [1]. Owing to these severe health implications and economic losses, more than 60 countries have embraced the need to regulate the aflatoxin content in foods. The aflatoxin export threshold per bag is 20 parts per billion (ppb) in the USA and 4 ppb in Europe [5]. As a result of this limitation, African countries’ export losses are about 670 million dollars per annum on the export of cereals, dried fruits, and nuts since they cannot meet the EU limitations [6]. Despite the efforts and regulations, 5 billion people worldwide are still at risk from aflatoxin exposure [7]. Therefore, there is a need to mitigate the aflatoxin problem by reducing the health risk and crop economic losses.

The aflatoxin-producing *A. flavus* fungus infects and causes aflatoxin contamination in several food crops, including maize and peanut, which are major sources of staple food globally, especially in Africa [8]. The United States peanut industry spends up to 58 million dollars annually to mitigate the effect of aflatoxin [9], making it an expensive problem for the agricultural industry. Developing countries contribute 60% of the global peanut production [10], and this makes aflatoxin production a significant threat to peanut production and consumption due to inadequate processing and storage facilities. Drought and high temperatures are reported to cause pre- harvest *A. flavus* infection [11]; however, drought-tolerant peanut lines are not necessarily associated with reduced *A. flavus* infection and aflatoxin contamination [12]. Effective implementation of post-harvest practices, such as drying, curing, and proper storage, can reduce aflatoxin contamination; nevertheless, effectiveness is based on obtaining peanut without pre-harvest infection [13]. For this reason, coming up with alternative strategies to reduce *A. flavus* infection and subsequent aflatoxin production is of great importance in the peanut industry. Current strategies deployed to manage *A. flavus* and aflatoxin contamination include chemical, biological, and cultural practices and identification of a stable source of resistance [14,15,16]. Previous studies have reported the use of alkali and oxidizing antifungal chemical agents to inhibit aflatoxin mold growth and subsequent aflatoxin contamination [17]. However, limitations, such as partial control, impairment of some food quality parameters, and the deposition of toxic compounds on peanuts, are the major drawbacks of the chemical agents [18]. The use of competitive atoxigenic fungal technology (CAFT) and promiscuous atoxigenic *Aspergillus* strains for biocontrol have reduced aflatoxin contamination levels in the field [11]. Nevertheless, CAFT increases the ability of molds to grow on peanuts, yielding low quality and poor peanut hygiene [11]. The discovery of host-pathogen resistance identification mechanisms, such as In-Vitro Seed Colonization (IVSC), Pre-harvest Aflatoxin Contamination (PAC), and Aflatoxin Production (AP), have been used to identify *A. flavus*-resistant peanuts [16]. Thus far, no single resistant germplasm line has been reported to all three host-pathogen resistance mechanisms due to inconsistent phenotyping [16]. On the other hand, the three host-pathogen mechanisms have independently identified some resistant and susceptible lines. 

Seeds obtained after shelling are dormant and do not possess active genetic resistance to combat pathogen infection. The peanut seed coat is the only protective layer of the cotyledons against *A. flavus* invasion after harvesting and shelling. The peanut seed coat comprises of five different cell layers at maturity, including the outer epidermal cells, spongy parenchyma, vascular bundles, inner epidermis, and perisperm [19]. These seed coat cell layers compress in preparation for dormancy, making it an impassable membrane. The outer epidermis of the seed coat is made up of a single layer of polygonal cells with thick cuticular walls with a wax layer in the junction between epidermal cells [20]. Genotypes with thicker seed coats, smaller hilum, and compact seed coat structures showed higher resistance to *A. flavus* infection [21,22]. In addition to the physical properties of the seed coat, the presence of secondary metabolites in the form of polyphenols has been reported in the peanut seed coat [23]. Polyphenols can be categorized into three different groups; these include phenolic acids, flavonoids, and coumarins [24]. Phenolic acids can be further divided into hydroxybenzoic and hydroxycinnamic groups, while flavonoids are made up of flavones, flavanones, flavonols, and flavanonols, isoflavones, flavanols, and anthocyanidins groups [24]. Cinnamic acid derivatives have been reported to possess antifungal activity against *A. flavus* in a yeast high-throughput bioassay [25]. This study aims at establishing the role of peanut seed coat and seed coat biochemicals in inhibiting *A. flavus* infection and colonization. The specific objectives are to (1) determine the response of peanut genotypes with intact seed coat to *A. flavus* infection; (2) measure the inhibitory effect of peanut seed coat biochemicals on *A. flavus* growth; (3) identify and quantify the individual biochemicals present in peanut seed coat and investigate their antifungal activities against *A. flavus* growth under in-vitro conditions; and (4) study the peanut seed coat development during seed development.

## 2. Materials and Methods

### 2.1. Isolation and Characterization of A. flavus Isolates from Peanut

Toxigenic *Aspergillus flavus* was isolated from infected peanut seeds. Fungal species’ isolation from seeds was performed by first surface sterilizing peanut seeds in 1.5% sodium hypochlorite, rinsing 3 times with sterile distilled water, blotting on a filter paper, and placing seeds in a 60-mm Petri dish. The Petri dishes were incubated in the dark at 28 °C for 3 days. Fungal accessions were sub-cultured to obtain toxigenic *A. flavus* and pure cultures. The pure cultures were stored at 28 °C for subsequent experiments. 

### 2.2. Morphological, Biochemical, and Molecular Characterization of A. flavus Isolates

*Aspergillus flavus* was identified based on colony characteristics and spore morphology [26]. Potato dextrose agar (PDA) and yeast extract sucrose agar (YESA) were supplemented with 0.3% β-cyclodextrin to a detect aflatoxigenic strains of *A. flavus* [27,28]. The toxigenic strain of *A. flavus* produces a characteristic blue fluorescence under UV light. The ammonium hydroxide test was also used to confirm the beta-cyclodextrin test [29]. For this, cultured plates were exposed to vapors generated by adding 2 mL of NaOH in a beaker. The bottom part of the plates was observed for subsequent color changes (Supplementary Figure S1). 

### 2.3. DNA Extraction and PCR Analysis

Molecular methods were employed to confirm the aflatoxigenic nature of the *A. flavus* isolates. DNA was extracted using Qiagen DNeasy plant mini kit following the manufacturers instruction. Markers representing the aflatoxin biosynthesis pathway genes in *A. flavus* were amplified by PCR. The following PCR conditions were employed: 94 °C for 5 min for initial denaturation step, followed by denaturation at 94 °C for 30 s; it was cooled at 55 °C for 1 min and was subjected to initial extension at 72 °C for 45 s and final extension for 7 min at 72 °C for 35 cycles. Gene-specific primers were designed using USDA batch primer 3 software (Table 1). 

### 2.4. Preparation of Fungal Cultures for Inoculation

Fungal culture was initiated by growing *A. flavus* culture in a Petri dish incubated at 28 °C. Inoculum was prepared by adding sterile distilled water to an 8-day old *A. flavus* culture in a Petri dish (28 °C). Spores were suspended in 10 mL of distilled water containing 1% of tween 80 (*v*/*v*). The suspension was filtered into a 50-mm Erlenmeyer flask using sterile cheesecloth and was stored at 4 °C in the dark. A hemocytometer was used to estimate the spore concentration and was adjusted to a final concentration of 1 × 10^7^ CFU mL^−1^ [30].

### 2.5. In-Vitro Seed Colonization (IVSC) Assay

Peanut seeds of varieties 55-437 and TMV-2 with and without seed coat were surface sterilized by dipping in 30 mL of 2% sodium hypochlorite for 3 min followed by three sterile-distilled water washes at 23–25 °C. Seeds were placed in a sterilized Petri dish with moist filter paper to provide enough moisture for fungal growth. Each seed in every Petri dish was inoculated with 10 µL of *A. flavus* spore suspension (1 × 10^7^ CFU mL^−1^) and incubated at 28 °C in the dark for 5 days. Inoculated seeds were observed for *A. flavus* colonization and growth daily. 

### 2.6. Estimation of A. flavus Growth in Inoculated Peanut Seeds

Peanut seeds used in the *IVSC* (55-437 and TMV-2 inoculated with aflatoxigenic *A. flavus*) were pooled together according to the respective genotype and crushed using a SPEX sample prep freeze mill 6870 (Metuchen, NJ, USA). DNA was extracted from the pooled sample using the Qiagen method. Quantitative PCR was carried out in a LightCycler 480 model II (Roche Diagnostics, Indianapolis, IN, USA) as described [31]. Briefly, reactions were prepared in 96-well plates (USA Scientific, Orlando, FL, USA) with a total reaction volume of 20 µL The reaction mix was prepared by adding 10 µL of 1 × IQ SYBR Green Supermix (Bio-Rad, Hercules, CA, USA) and 0.6 µL of forward (AFITS-F: CGTCATTGCTGCCCATCAAG) and reverse (AFITS-R: ATCCCTACCTGATCCGAGGT) primers at a concentration of 200 nM each, 0.37 µL of 50 ng template DNA, and 8.43 µL of nuclease-free water specifically for *Aspergillus flavus ITS* gene (KX610727.1), which amplified the *A. flavus* fungal DNA. 

Each DNA sample was loaded in 6 replicates in a 96-well plate. The temperature profile for qPCR was as follows: 95 °C for a 5 min denaturation step, followed by 40 cycles of amplification at 95 °C for 10 s and 65 °C for 35 s, and melt curve analysis of heating to 95 °C, cooling to 65 °C for 1 min, and heating to 97 °C at a rate of 1 °C/5 s. The primer pair AHSSP-F: CCACCGTCTGAACAGTCTCT and AHSSP-R: CCCGCAAAAGGTTTTCTCCA was designed to specifically amplify the *Arachis hypogea* gene (AY439332.1). Primers were used at a concentration of 300 nM for gene amplification of AY439332.1. The absolute quantification module of the LightCycler software was used to determine Cq and pathogen copy number [31]. The threshold Cq value was manually adjusted to 1.0 for all qPCR experiments to enable run-to-run comparisons. DNA melt peaks were analyzed at the end of each run to ensure specificity of amplification of the β-tubulin gene fragment.

### 2.7. Chemical Reagents 

Chemicals used for HPLC (methanol and acetic acid) and solid-phase extraction (sodium bicarbonate) were all analytical grade and were purchased from Fisher Scientific (Hampton, NH, USA). Water used for the experiments was obtained from a Milli-Q purification system in the lab (Millipore, Milford, MA, USA). Standards of phenolic acids, protocatechuic, p-hydroxybenzoic, vanillic, caffeic, gallic acid *p*-coumaric, and ferulic, were purchased from Fisher Scientific (Hampton, NH, USA).

### 2.8. Extraction of Soluble and Insoluble Phenolic Compounds from Peanut Seed Coats

Seed coats were removed from 55-437 and TMV-2 by hand peeling. The collected seed coats were ground into powder using a SPEX sample prep freeze mill 6870 (Metuchen, NJ, USA). The freeze mill condition was set to three cycles for grinding samples at 10 CP speed. Briefly, samples were pre-cooled in liquid nitrogen for 7 min and ground for 2 min and re-cooled in liquid nitrogen for 2 min. The powdered samples were stored at 4 °C until further use. Triplicates of the powdered seed coat samples (1 g each) of 55-437 and TMV-2 were added to individual 15-mL capped centrifuge tubes. Samples were extracted twice using 10 mL of acetone/water/acetic acid (70:29.5:0.5 *v*/*v*/*v*) [32]. The mixture was extracted for 3 h under light using an orbital shaker followed by 12 h shaking in the dark at 300 rpm. The extract was centrifuged at 2000× *g* for 10 min and collected in a new centrifuge tube. The extracts were combined, and this accounted for the total soluble phenolic compounds fraction and was stored at 4 °C in the dark until further use. The remaining crude extracts were air-dried for cell-wall-bound phenolic extraction. Cell-wall-bound phenolics were extracted with 2 mL of 2 N NaOH in a water bath at 60 °C for 30 min with intermittent shaking. The solution was then acidified with 2 mL of 2 N HCL. The mixture obtained was extracted with 10 mL of acetone/water/acetic acid (70:29.5:0.5 *v*/*v*/*v*) at 4 °C. The extract was centrifuged at 2000× *g* at 10 min at 4 °C to obtain the wall-bound phenolic compound fraction. The wall-bound phenolic compounds were stored at 4 °C in the dark until further use. Both soluble and insoluble phenolic compounds obtained from the seed coat were dried in a rotary evaporator to remove all the extraction solvents (acetone, acetic acid, and water) from the phenolic compounds. The rotary evaporator water bath was set at 40–45 °C at a speed of 30–40 rpm. The dried soluble and insoluble phenolic compounds were stored in an amber bottle at 4 °C.

### 2.9. Radial Growth Bioassay of Seed Coat Extracts Obtained from Peanut 

PDA medium with 0.25 mg/mL chloramphenicol was used for this experiment. Sterile Petri dishes were placed in a sterile laminar flow hood; two perpendicular straight lines were drawn on the bottom of each Petri dish. The dried soluble phenolic compounds were first dissolved in DMSO, which is the best extract solvent for the radial growth bioassay [33]. With the help of a pipette, 2 mL of dissolved soluble phenolic compound extracted from 55-437 or TMV-2 was added to a 50-mL falcon tube containing 35 mL of PDA media (triplicates). The PDA medium containing the soluble phenolic compound was mixed well and gently poured into a Petri dish and allowed to solidify. The control plate was prepared by adding 35 mL of PDA medium dissolved in 2 mL DMSO. Each Petri dish was inoculated with 10 µL of *Aspergillus flavus* spore suspension (1 × 10^7^ CFU mL^−1^). The Petri dish was gently covered, and the edges of the plate flamed, sealed, and incubated at 28 °C. The inoculated medium was observed for fungal growth daily. Radial growth bioassay data were taken until the control plate was full. The area under the disease progress curve (AUDPC) was computed for each PDA medium with seed coat extract using the formula as described by Shaner and Finney [34]:

$$\mathrm{AUDPC}=\sum_{i}^{n-1}\left(\frac{y_{i}+y_{i+1}}{2}\right)\left(t_{i+1}-t_{i}\right)$$

The percentage of *A. flavus* inhibition for insoluble seed coat extract was calculated using the formula as described by Bekker et al. [35]:

$$I=\left(\frac{C-T}{C}\right)\times 100$$

### 2.10. Solid Phase Extraction and HPLC Analysis of Peanut Seed Coat Phenolic Acids 

After extraction and drying in the rotary evaporator, the soluble extract obtained from peanut seed coats (55-437 and TMV-2) was dissolved in solvent prepared by mixing 2 mL of methanol and distilled water solution in 1:1 (*v*/*v*). To dissolve the soluble extract, 1 mL of TCMA (3-4-5, trimethyoxy cinnamic acid) was added as an internal standard. These extracts were loaded on top of waters Sep-Pak C-18 SPE cartridges (500 mg, 6 mL), which were preconditioned with 10 mL of methanol followed by 10 mL of distilled water. The primary eluent obtained after washing was made of a complex of polar phenolics and was adjusted to a pH of 7.0–7.2 using a 5% sodium bicarbonate aqueous solution to make it anionic. A waters Sep-Pak NH_2_ SPE cartridge (1 g, 6 mL) was preconditioned with 10 mL of distilled water followed by 20 mL of 0.1% sodium bicarbonate aqueous solution before the anionic primary eluent was passed through the NH_2_ cartridges. The sorbent beds were dried for one min under vacuum, and the analytes of interest were washed using 3 mL of 70% methanol solution. The eluates were analyzed using HPLC (high performance liquid chromatography). An Agilent 1100 (Agilent Technologies, Santa Clara, CA, USA) series LC system equipped with a degasser, binary pump, autosampler, column compartment, and UV-DAD was used for this analysis. A total of 10 µL of the eluates was injected into the column by the autosampler. The separation of phenolic acids was performed on an Agilent reverse-phase C-18 column (5 µm × 4.6 × 150 mm) at a flow rate of 0.5 mL min^−1^. The mobile phase consisted of 0.25% acetic acid in deionized water at a pH of 2.3 (Mobile phase A) and methanol (Mobile phase B). The analysis was performed using a binary gradient system. The gradient profile is as follows, 0–2 min 85% A, 15% B; 2–10 min 75% A, 25% B; 10–15 min, 72.5% A, 27.5% B; 15–35 min 70% A, 30% B; 35–53 min 65% A, 35% B; 53–61 min 60% A, 40% B; 61–70 min 50% A, 50% B; 70–75 min 40% A, 60% B, 75–79 min 30% A, 70% B; and 79–87 min 95% A and 5% B. The analytes were detected at 280 nm, and separated components were identified by matching UV spectra and retention time (RT) mapping, with authentic standards. A known concentration of the (TCMA) internal standard was added to each external standard. The peak area of each external standard and internal standard was obtained after each HPLC run; this was repeated for three technical replicates. The peak area was used to construct calibration curve for each external standard. The calibration curve obtained was used to quantify each phenolic acid present in peanut seed coat (Table 2). The method was validated by calculating the LOD and LOQ of each external standard. The limit of detection (LOD) and limit of quantification (LOQ) calculation was based on the standard deviation of the intercepts (σ) and the average slope from the three regression lines (*S*) using the following equations: LOD = (3.3 × σ)/*S* and LOQ = (10 × σ)/*S*.

### 2.11. Antifungal Activity Assay of the Phenolic Acids 

Six phenolic acids that were identified in the peanut seed coat (gallic acid, protocatechuic acid, vanillic acid, syringic acid, *p*-coumaric acid, and ferulic acid) were screened for their antifungal activity using radial growth assay. Each compound was weighed and was dissolved in DMSO at four different concentrations (0.667, 0.333,0.1667, and 0.033 mg/mL). One mL of each compound was dissolved into a 50-mL falcon tube containing 35 mL of PDA medium (for all four concentrations in triplicates). The medium containing each compound at a particular concentration was gently poured into a Petri dish and allowed to solidify. The control used for this experiment contained 35 mL of PDA medium mixed with 1 mL of DMSO. Each Petri dish was inoculated with 10 µL of spore suspension (1 × 10^7^ CFU mL^−1^) of *A. flavus*. The Petri dish was gently covered, and the edges of the plate were flamed, sealed, and incubated at 28 °C. Fungal growth was observed daily, and the inhibitory effect of each phenolic compound at the four different concentrations were determined using the formula of Bekker, Kaiser, vd Merwe, and Labuschagne [35].

### 2.12. Determination of Total Lignin Content and Lignin Monomer Unit Contents of Peanut Seed Coat 

The remaining crude fraction of seed coat obtained after soluble and wall-bound phenolic extraction was used to quantify the total lignin content. The lignin content was determined using the Thioglycolic acid (TGA) method as described previously [31]. Briefly, 1 mL of TGA reagent was added to the crude fraction and incubated at 80 °C for 3 h to facilitate the solubility of lignin. After incubation, the tubes were centrifuged at 2000× *g* for 10 min, and the supernatant was discarded. The pellet obtained was then washed with 1 mL of double-distilled water followed by centrifugation, and the supernatant was discarded. The pellet was vacuum dried and incubated with 1 mL of 1 M sodium hydroxide on a thermal shaker at 37 °C for 24 h and centrifuged. The supernatant was collected, acidified with 0.2 mL of concentrated HCl, and incubated at 4 °C for 4 h to extract all possible amounts of the derivatized lignin. After incubation, the tube was centrifuged, vacuum dried, and dissolved in dimethyl sulfoxide (DMSO) to estimate lignin content using UV absorbance reading at 280 nm. The internal standard used was an industrial bamboo lignin obtained from sigma Aldrich (St. Louis, MO, USA).

Individual monomers of lignin were determined using the thioacidolysis procedure [36]. For each sample, 2.5 mg of seed coat powder was weighed into a 5-mL reaction glass vial mixed with 250 µL of a reaction mixture (2.5% boron trifluoride etherate and 10% ethanethiol, in dioxane (*v*/*v*)) added to each vial, and air was purged with nitrogen gas before sealing. The vials were incubated in a heating block for 4 h at 100 °C with intermittent shaking. The remaining procedure was followed as described [36], except using ethyl acetate instead of mercuric chloride. Gas chromatography was performed using an Agilent GC 7890A coupled with an Agilent MS 5975C with a triple-axis detector. An Agilent 30 m by 0.250 mm internal capillary column was used for this experiment. Injections of 1 µL were separated using helium gas as a carrier at 1 mL per min speed. Inlet and detector temperatures were set to 250 °C, while the oven temperature profile was set at 200 °C, for 1 min, for 215 °C for 1 min, followed by a gradient increase in temperature to 275 °C for 1 min, and was finally held at 250 °C for 5 min and 200 °C after the end of the run. 

### 2.13. Quantification of Soluble and Insoluble Tannin Present in Peanut Seed Coat

Frozen, ground tissue of 10 mg was added to 1.5 mL centrifuge tubes with 1 mL of 70% acetone/0.5% acetic acid and was extracted for 30 min in the dark at 4 °C. The solution was centrifuged at 2500× *g* for 10 min. The supernatant was collected, and the extraction was repeated three times. The supernatants were pooled together and were adjusted to a total of 4 mL. The pellet was stored and used for insoluble proanthocyanidin analysis. Three mL of diethyl ether was added and vortexed, and the solution was allowed to separate into phases in a −20 °C freezer. The bottom fraction (770 µL) was collected using a syringe for soluble proanthocyanidin analysis (PA). Half (385 µL) of the soluble proanthocyanidin was taken, and an equal volume of methanol and 19 µL of 2% DMACA solution was also added and kept at room temperature for 15 min. Soluble PA was measured at an absorbance at 640 nm using a spectrophotometer. For insoluble PA quantification, 1% SDS and 1.2 mL of 19:1 butanol-hydrochloric acid was added to the pellet. The solution was incubated at 95 °C for 90 min to dissolve, and the mean absorbance was measured at 555 nm using a spectrophotometer [37].

### 2.14. Gene Expression Analysis of Lignin- and Tannin-Related Genes in Peanut Seed Coat 

Total RNA was extracted from the seed coat at the R6 stage using Spectrum^TM^ Plant Total RNA kit (Sigma-Aldrich, St. Louis, MO, USA). Several genes important in the biosynthesis of both PAs and lignin were selected based on the Arabidopsis gene orthologs of peanut using PeanutBase (https://www.peanutbase.org, accessed on 9 February 2017) (Table 3). Genomic DNA from RNA sample was removed by on-column DNase I digestion (Sigma-Aldrich, St. Louis, MO, USA). The first strand of cDNA was synthesized using 0.5–1 µg DNA-free total RNA with the iScript^TM^ Reverse Transcription Supermix RT- qPCR kit (Bio-Rad, Hercules, CA, USA). Complementary DNA (cDNA) was diluted to 20-fold with water and used for q-PCR using FastStart Essential DNA Green Master kit (Roche, IN, USA). LightCycler 96 (Roche, IN, USA) was used to perform the qPCR experiment and results were calculated using ΔΔCt method [38].

### 2.15. Histological Analysis of Peanut Seed Coat Structure at the Developmental Stage

For light microscopy, a 1 mm × 1 mm section of the seed coat cut directly from fresh seeds collected from the field at different developmental (R3 to R7) stages were used [39]. The sectioned tissue was fixed using a mixture of glutaraldehyde, in 0.1 M PO_4_ buffer, (pH 7) and osmicated using osmium tetroxide [40] with some modifications using 2% glutaraldehyde and 2% osmium tetroxide. For transmission electron microscopy, the sections were fixed in a mixture of glutaraldehyde, paraformaldehyde, and cacodylate buffer, with pH 7 as described by Beeckman et al. [41]. The samples were washed five times with deionized water followed by dehydration with increasing percentage ethanol washes. The tissue for both light microscopy and transmission electron microscopy were embedded in epoxy resin and transferred to a 1:1 mixture of acetone: epoxy for 1 h at 20 °C, moved to a 1:2 mixture for 1 h at 20 °C, then a 1:4 mixture for 1 h, and was finally kept in pure epoxy overnight. The tissue was cut with a microtome and observed under light stained with 1% (*w*/*v*) toluidine blue O in 1× (*w*/*v*) sodium borate (pH 11) for the light microscopy. The sections were examined using Olympus S761 (Waltham, MA, USA). Photographs were taken using the Olympus software imaging solutions. For TEM, the sections were examined using Hitachi H-8100 scanning transmission electron microscope (Schaumburg, IL, USA). The light microscope was used to investigate the various types of cells that form the seed coat. Transmission Electron Microscopy (TEM) was used to gain more insight into the components of the outer epidermal cell structures. 

### 2.16. Statistical Analysis

The data obtained were subjected to analysis of variance (ANOVA) using Genstat 12th Edition. The post-hoc Tukey’s test analysis was used to test the significant difference of different groups for most of the experiments. Using Microsoft excel, the Students *t*-test (one tail and type 2) was used to calculate the significant difference between quantified phenolic compounds from 55-437 and TMV-2. 

## 3. Results

### 3.1. Identification and Characterization of Toxigenic A. flavus Isolate 

The *A. flavus* strain used was isolated from infected peanut seeds and characterized for the experiments in the present study. Eight *A. flavus* isolates were identified macroscopically based on their characteristic greenish color. Based on the cultural method of detecting toxigenic strains (Table 4), seven out of eight *A. flavus* isolates were identified to be aflatoxigenic except isolate 5. Yeast extract sucrose agar (YES) (with or without beta cyclodextrin) and PDA (with beta-cyclodextrin) were used to distinguish toxigenic strains from atoxigenic strains (Table 4). The toxigenic strain was further confirmed by amplifying aflatoxin producing genes. The Polymerase Chain Reaction (PCR) data showed that *A. flavus* isolates 1, 2, and 3 contain all the tested toxigenic biosynthetic genes (Table 5). Isolate 6 and 4 showed five out of six biosynthetic genes present except for *afl*M gene and *afl*D, respectively. Isolate 7 showed the presence of four biosynthetic genes, and isolate 8 showed two biosynthetic genes. Isolate 5, on the other hand, showed absence of all the biosynthetic genes except *afl*R gene, which is consistent with the atoxigenic nature of the strain (Table 5). Together, isolate 1, 2, and 3 were characterized as toxigenic strains, while isolate 5 was categorized as an atoxigenic strain. Toxigenic and atoxigenic isolates were used in the experiments in this study as required.

### 3.2. Intact Seed Coat Reduces the A. flavus Infection

**Figure 1:**
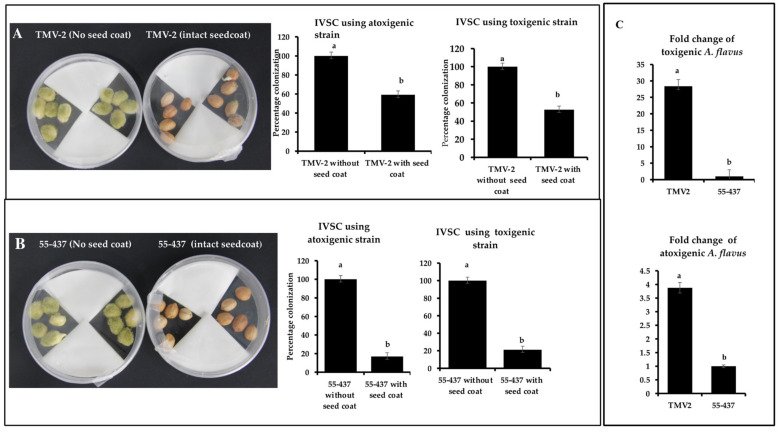
In-vitro seed colonization assay of peanut lines 55-437 and TMV-2 with and without seed coat. Sterilized peanut seeds of TMV-2 (**panel** (**A**)) and 55-437 (**panel** (**B**)) with intact seed coat and without seed coat inoculated with aflatoxigenic (isolate 3) and non-aflatoxigenic (isolate 5) *A. flavus* strains. Percentage of colonization was calculated as (number of seeds colonized by the pathogen)/(total number of seeds) × 100. The data is an average of three technical replicates and three biological replicates. The fungal abundance of toxigenic and atoxigenic *A. flavus* strains was estimated using qPCR (**panel** (**C**)). Peanuts from TMV-2 and 55-437 with intact seed coat obtained after IVSC assay was ground and DNA was isolated from the pooled samples and used for relative quantification using qPCR. Fold change for the fungal abundance for toxigenic (upper panel) and atoxigenic (lower panel) stains was calculated from the qPCR data. One-way ANOVA was used to determine the significant difference between different groups. Means not sharing the same letter are significantly different (*p* < 0.05). The error bar represents the standard error (*n* = 9).

To test the concept of the physical barrier function of the seed coat, IVSC with and without seed coat was performed. IVSC was performed using resistant (55-437) and susceptible (TMV-2) peanut accessions with toxigenic and atoxigenic strains. The percentage incidence of toxigenic *A. flavus* strain on 55-437 and TMV-2 with intact seed coat was 21.20% and 52.59%, respectively, while atoxigenic strain incidence was 16.89 and 59.52% on 55-437 and TMV-2, respectively (Figure 1A,B), on the fifth day post-inoculation. For peanuts without seed coats, the percentage colonization after the first day of inoculation was 50% for both TMV-2 and 55-437 and showed a rapid increase and reached 100% on third day in both TMV-2 and 55-437 seeds. The relative abundance of toxigenic and atoxigenic strains of *A. flavus* isolates was determined using a quantitative PCR (qPCR) approach in the intact seed coat IVSC experiment (Figure 1C). The fold change of toxigenic isolate was 27 times more in TMV-2 than 55-437, while fold change of atoxigenic isolate was 3.5 times more in TMV-2 than 55-437. Overall data showed that lack of intact seed coat results in rapid infection by *A. flavus*. 

### 3.3. Peanut Seed Coat Phenolic Extracts Inhibited A. flavus Growth 

**Figure 2:**
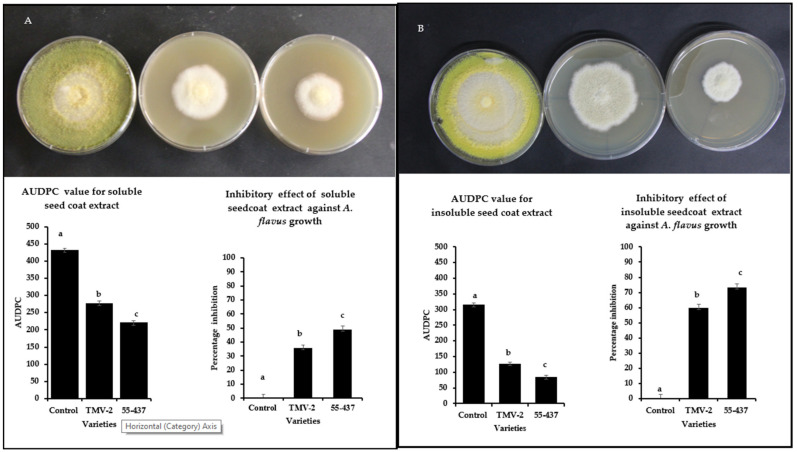
Radial growth bioassay of extracted soluble and insoluble peanut seed coat biochemicals from TMV-2 and 55-437. PDA media plates containing seed coat biochemicals extracts were inoculated with 10 µL of *A. flavus* spores (1 × 10^7^ CFU mL^−1^) and incubated at 28 °C. Radial growth was measured and used to calculate the Area Under Disease Progress Curve (AUDPC) and the percentage of inhibition over seven days. One-way ANOVA was used to determine the significant difference between different groups. Means not sharing the same letter are significantly different (*p* < 0.05). The error bar represents the standard error (*n* = 9). Radial growth of PDA media-containing soluble (**panel** (**A**)) and insoluble (**panel** (**B**)) biochemical extracted from peanut seed coat of DMSO control (left), TMV-2 (middle), and 55-437 (right) PDA media containing.

Intact seed coat experiments showed a stronger inhibition of *A. flavus* growth in resistant line (55-437) compared to susceptible (TMV-2) line (Figure 1). In addition to the physical barrier, it is possible that biochemicals in the seed coat can inhibit the *A. flavus* growth. To test this hypothesis, soluble and insoluble crude extracts were extracted from the peanut seed coats (55-437 and TMV-2) and tested their inhibitory effect using a radial growth bioassay (RGA). RGA assay showed significantly higher inhibition in the PDA media with seed coat extract for 55-437 compared to TMV2 and Dimethylsulfoxide (DMSO) control (Figure 2). The Area Under Disease Progress Curve (AUDPC) value for soluble seed coat extract of 55-437 (220) was significantly lower compared to PDA medium with TMV-2 extract (277). The AUDPC value of the DMSO control was about twice (430) the value recorded for 55-437 and TMV-2. The percentage inhibition for seed coat soluble extract for 55-437 (48.8%) was significantly higher than seed coat extract for TMV-2 (35.6%). In terms of effect of insoluble seed coat on fungal growth, the AUDPC value observed for PDA media amended with 55-437 (84) and TMV-2-extracts (126) were less than half the AUDPC value recorded for both soluble extracts. The percentage inhibition for seed coat insoluble crude extract for 55-437 (73.29) was significantly higher than the insoluble seed coat extract of TMV-2 (59.87). This indicates that insoluble biochemical extract contribute more to the regulation of *A. flavus* growth in peanut than soluble biochemical extract.

### 3.4. Identification and Quantification of Phenolic Acids in the Peanut Seed Coat 

**Figure 3:**
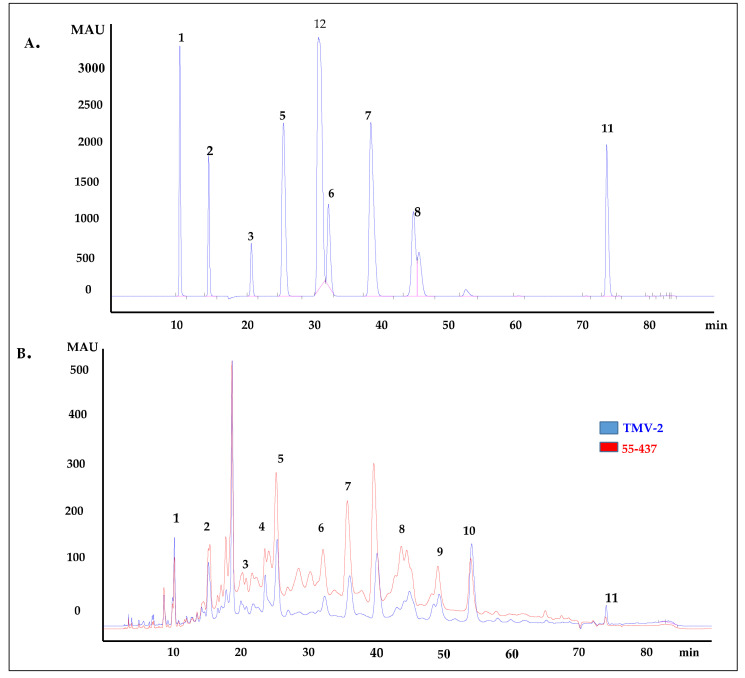
HPLC chromatogram of peanut seed coat phenolic acids. (**A**) HPLC chromatogram of external standards used to identify the phenolic acids present in peanut seed coat at 280 nm. Peaks: (1) gallic acid, (2) protocatechuic acid, (3) 4-hydrobenzoic acid, (5) vanillic acid, (6) syringic acid, (7) *p*-coumaric acid, (8) trans-o-coumaric acid, (11) 3,4,5 trimethoxycinnnamic acid (Internal standard), and (12) vallin. (**B**) HPLC chromatogram of extracted phenolic acids from 55-437 and TMV-2 peanut seed coat by SPE C-18 and NH2 cartridges (detection at 280 nm). The peaks were identified by comparing the retention time of external standard to peaks of the analyte. Peaks: (1) gallic acid, (2) protocatechuic acid, (3) 4-hydrobenzoic acid, (4) caffeic acid, (5) vanillic acid, (6) syringic acid, (7) *p*-coumaric acid, (8) trans-o-coumaric acid, (9) ferulic acid, (10) sinapinic acid, and (11) 3-4-5, trimethoxycinnamic acid (internal standard).

**Figure 4:**
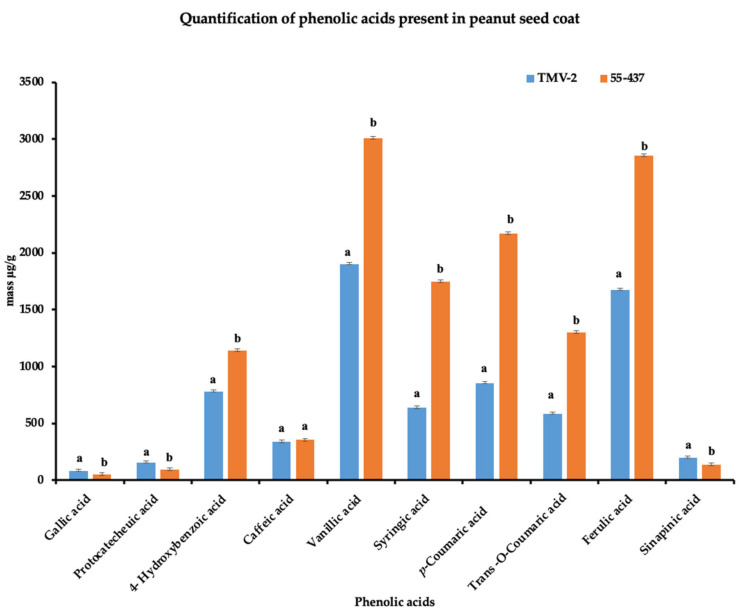
Quantification of peanut seed coat phenolic acids. Phenolic acids isolated from TMV-2 and 55-437 seed coats by SPE C-18 and NH2 cartridges (detection at 280 nm). Quantification was performed using the calibration curve. The quantified data are an average of 3 technical replicates and 3 biological replicates. The Students *t*-test was used to determine the significant difference between different groups. Means not sharing the same letter are significantly different (*p* < 0.05), and error bars represent standard error (*n* = 9).

Since the seed coat soluble and insoluble extracts inhibited *A. flavus* growth, the HPLC was used to identify and quantify the individual phenolic compounds (biochemicals) present in 55-437 and TMV-2 peanut seed coat crude extracts. The HPLC chromatogram showed twelve (12) different peaks in 55-437 and TMV-2 seed coats extracts and, using external standards, 10 peaks were identified as phenolic acids with two identified as unknown compounds (Figure 3B). Five of them are hydroxybenzoic derivatives (gallic acid, protocatechuic acid, 4-hydroxybenzoic acid, caffeic acid, and vanillic acid), and the remaining phenolic acids are hydroxycinnamic derivatives (syringic acid, *p*-coumaric acid, trans-o-coumaric acids, ferulic acids, and sinapinic acids). Comparative analysis showed a significant difference between 55-437 and TMV-2 for each identified phenolic compound (*p* < 0.05). Overall, 55-437 showed a higher amount of phenolic acid present in its seed coat compared to TMV-2 seed coat with the exception of gallic acid, protocatechuic acid, and sinapinic acid (Figure 4). Vanillic acid is the most abundant phenolic acid present in the seed coat of 55-437 (3011.16 µg/g) and TMV-2 (1899.57 µg/g). On the other hand, gallic acid was the least abundant compound in both 55-437 (49.47 µg/g) and TMV-2 (79.41 µg/g). The data show that the type and amounts of phenolic acids deposited in the peanut seed coat differ between the two varieties. 

### 3.5. Hydroxycinnamic Acid Derivates Inhibits A. flavus Growth on PDA Media 

**Figure 5:**
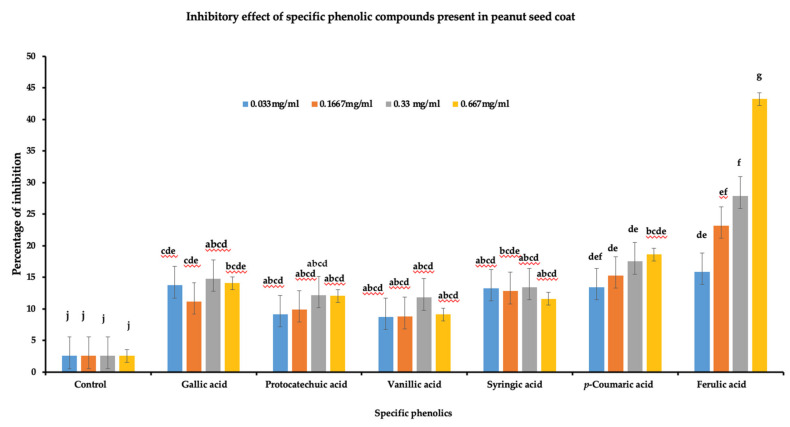
Inhibitory effect of phenolic acids against *A. flavus* growth. Six individual phenolics were dissolved in DMSO at four different concentrations (0.667 mg/mL, 0.33 mg/mL, 0.1667 mg/mL, and 0.033 mg/mL). Individual phenolic acids dissolved in DMSO were added to PDA media along with control DMSO, and plates were inoculated with 10 µL of *A. flavus* spores (1 × 10^7^ CFU mL^−1^). The Petri plate was incubated at 28 °C, daily radial growth was measured for seven days, and the data were used to calculate the percentage of inhibition (3 biological replicates). One-way ANOVA was used to determine the significant difference between different groups. Means not sharing the same letter are significantly different (*p* < 0.05), and the error bar represents the standard error (*n* = 9).

To determine the role of the identified phenolic compounds in *A. flavus* growth inhibition, a radial growth bioassay was done using six phenolic compounds (gallic acid, protocatechuic acid, vanillic acid, syringic acid, *p*-coumaric acid, and ferulic acids). Individual phenolic acid compounds at different concentrations were added to PDA plates for radial growth assay. A significant reduction in the *A. flavus* growth (*p* < 0.05) was observed for all the tested phenolic compounds at the four different concentrations (Figure 5). Among the six phenolic acids, ferulic acid showed the highest level of inhibition at 0.667 mg/mL (43.26%), followed by *p*-coumaric acid at 0.667 mg/mL (18.27%), while vanillic acid showed lowest inhibition compared to other phenolic acids at 0333 mg/mL (8.75%). Furthermore, in terms of concentration, ferulic acid and *p*-coumaric acid showed a linear increase in percentage of inhibition with ferulic acid concentration. On the other hand, there was no linear response in the percentage of inhibition of vanillic acid, protocatechuic acid, gallic acid, or syringic acid to increased concentration. Overall, the data indicate that phenolics acids play a role in *A. flavus* growth inhibition and could be a contributing factor in differentiating between resistant and susceptible lines against *A. flavus* pathogen.

### 3.6. Resistant and Susceptible Lines Showed Differences in Tannins and Lignin Monomer Composition in Peanut Seed Coat 

**Figure 6:**
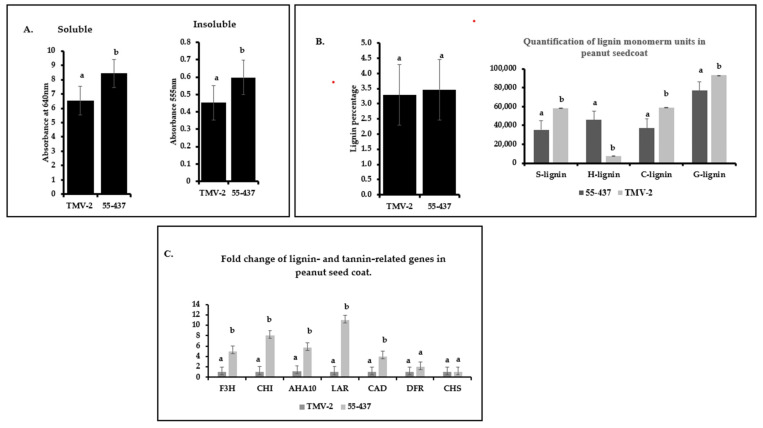
Quantification of insoluble and soluble proanthocyanidin in peanut seed coat. (**A**) Seed coats from greenhouse grown peanut varieties 55-437 and TMV-2 was used for biochemical analysis of soluble and insoluble proanthocyanidins. The error bar represents the standard error. The data are an average of three technical replicates and three biological replicates. One-way ANOVA was used to determine the significant difference between different groups. Means not sharing the same letter are significantly different (*p* < 0.05). (**B**) Cell-wall lignin was extracted from the collected tissue and was measured using the spectrophotometer. Lignin monomer units were estimated using GC-MS. The data are an average of three technical replicates and three biological replicates. One-way ANOVA determined the statistically significant difference between different groups. Means not sharing the same letter are significantly different (*p* < 0.05), and the error bar represents the standard error. (**C**) Peanut varieties 55-437 and TMV-2 were grown in the green house, and seed coat tissue was harvested 67 days after planting. The seed coat was used for RNA extraction and qPCR analysis. Statistically significant difference between different groups was determined by one-way ANOVA. Means not sharing the same letter are significantly different (*p* < 0.05).

In addition to phenolic compounds, phenolic polymers, such as tannin and lignin, are also known to confer disease resistance. Spectrophotometer analysis of proanthocyanidins showed difference between 55-437 and TMV-2 (Figure 6a). Both soluble and insoluble proanthocyanidin were significantly (*p* < 0.05) higher in 55-437 compared to TMV-2 (Figure 6a). The total lignin content in the peanut seed coat did not show significant difference between 55-437 and TMV-2 (Figure 6b); however, there was a significant (*p* < 0.05) difference between TMV-2 and 55-437 for all the monomer units (Figure 6b). The guaiacyl, sinapyl, and catechyl monomers were higher in TMV-2 than 55-437, while p-hydroxyphenyl monomer, on the other hand, was six times higher in 55-437 than in TMV-2. 

### 3.7. Phenylpropanoid Related Genes Showed Differential Expression Pattern between TMV-2 and 55-437

Since TMV-2 and 55-437 showed differences in the phenolic compounds and lignin monomer composition, the expression of seven phenyl propanoid pathway genes were investigated at the R6 stage of the peanut seed coat (Figure 6c). Among the proanthocyanidin production genes, the *LAR* (leucoanthocyanidin reductase) had a ten-fold higher expression in 55-437 compared to TMV-2, followed by the Chalcone isomerase (*CHI*) gene, which had seven-fold higher expression in 55-437 than TMV-2. The flavanone-3-hydroxylase (*F3H*) gene had four-fold higher expression in 55-437 compared to TMV-2, while there was no significant difference between 55-437 and TMV-2 in dihydroflavanol-4- reductase (*DFR*) and Chalcone synthase (*CHS*) genes. The Cinnamyl alcohol dehydrogenase gene (*CAD*) showed a significant difference with three-fold higher expression in 55-437 compared to TMV-2. Lastly, the autoinhibited H (+)-ATPase Isoform 10 (AHA10) gene showed three times higher expression in 55-437 compared to TMV-2. 

### 3.8. Peanut Seed Coat Developmental Biology Studies Showed Thick Outer and Innermost Layers with Compressed Inner Layers

**Figure 7:**
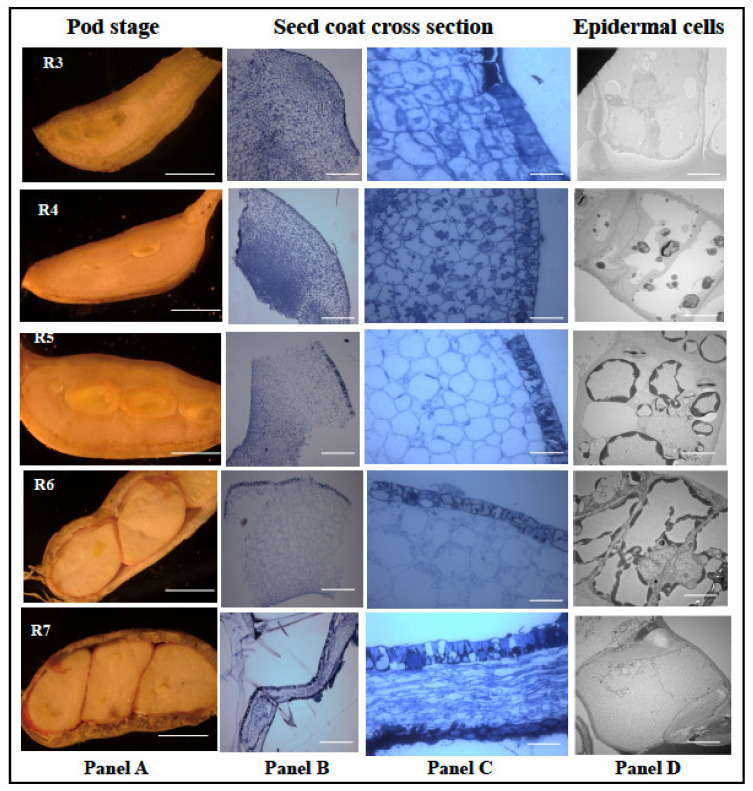
Histological analysis of peanut seed coat different developmental stages. Peanut plants were grown in the greenhouse; the seeds were harvested at 31 Days After Planting (DAP) to obtain the R1 stage, 39 DAP for R2 stage, 46 DAP for R3 stage, 52 DAP for R4 stage, 57 DAP for R5 stage, 67 DAP for R6 stage, and 80 DAP for R7 stage. The seed coat tissues obtained from the harvest were treated and viewed under the light and electron microscopes. (**A**) Longitudinal cross-section of peanut under different reproductive stages. (**B**) The treated seeds viewed at 120× zoom and (**C**) at 600× zoom. (**D**) The epidermal cells of the peanut seed coat viewed at 400× zoom. Scale bar: Panel A, 2 mm; Panel B, C, 2 μm; and D, 0.2 μm.

Since peanut seed coat plays a significant role in physical and biochemical barrier against *A. flavus* infection and growth, it is important to understand the developmental biology of the seed coat. Bright light and transmission electron microscopes were used to study the developmental series (R3 to R7) of peanut seed coat. The first evidence of seed formation is first seen at the R3 stage (Figure 7). At this stage, the pod is made of spongy parenchymous tissues (Figure 7), and at the R4 stage, the seed coat and the cotyledon begin to form with distinct, thick outer and innermost layers. Distinct punctate structures are seen in all the cell layers at the R4 stage (panel C), and at the R5 stage, the cell size had increased, the punctate structures were reduced in the parenchymatous cells and increased in the epidermal cells (panel C). The TEM micrographs showed an increase in deposition of unknown compounds in vacuoles (panel C; R4, R5, and R6). Towards the R6 (whole seed) stage, the cotyledon occupied most of the pod volume. The light micrograph revealed compression of inner seed coat layers, while inner and outer layers remained intact. 

## 4. Discussion

Aflatoxin contamination due to *Aspergillus* species is a major cause of economic loss in several crops, such as corn, cotton, soybean, peanuts, and tree crop products [9], causing losses of U.S. 52.1 million to U.S. 1.68 billion annually in the U.S. [42]. In addition to the economic impact, it was estimated that aflatoxin is responsible for 4.6 to 28.2% of global hepatocellular carcinoma cases [43]. Several strategies, such as chemical, biological, and cultural practices and development of resistant germplasm lines, have been deployed [14,15,16]; however, the aflatoxin problem is still a major issue in several crop plants. Use of chemical agents, such as synthetic fungicides, to control *A. flavus* infection and aflatoxin contamination not only results in the deposition of toxic compounds in peanuts but also poses the issue of *A. flavus* developing resistance to chemicals [44]. Although biological control, such as CAFT, provides a milder effect on peanuts in terms of toxic material deposition, its potency to trigger accumulation of other secondary metabolites poses food quality and safety issues [45]. Developing genetic resistance in peanut varieties is an ideal strategy, as it protects the peanuts both at pre- and post-harvest stages. Several lines have been identified and characterized to be *A. flavus* resistant; however, inconsistent phenotyping is a major constraint in identification of a stable source of resistant line [16]. Hence, developing novel sources of heritable resistance and germplasm lines is crucial to address *A. flavus* infection and aflatoxin contamination. 

The *IVSC* assay for *A. flavus* resistance exploits the seed-coat-mediated physical and biochemical barrier both at pre- and post-harvest stages. The present study establishes that the seed coat is important for inhibiting *A. flavus* infection and growth. The lower percentage of seed colonized by *A. flavus* in both 55-437 and TMV-2 with intact seed coats may be attributed to the physical and/or biochemical nature of the seed coats, as the seed coat is primarily composed of cell walls. Previous studies used to distinguish resistant and susceptible lines using IVSC proposed that the observed resistance could be attributed to seed coat thickness or permeability [46]. The developmental biology data showed that the seed coat is composed of three layers at maturity: the thick epidermis layer, the spongy compressed parenchymal layers, and the inner thicker epidermis. These seed coat layers appear to form a formidable structural barrier against *A. flavus* infection and growth. Further, the outer epidermis of the peanut seed coat has been reported to be made up of insoluble mixtures of wax and fatty acids, which makes it heavily cutinized [47]. It was also reported that the size of the hila and amount of wax plays a role in *A. flavus* infection, with resistant lines having smaller hila, making it difficult for *A. flavus* to invade [48]. Overall, the present and previous investigations establish that seed coat acts as a physical barrier against *A. flavus* infection.

Similarly, biochemical compounds, such as phenolic compounds produced by plants, are known to possess antimicrobial [49] as well as antioxidant [50] properties. Presence of phenolic compounds has been reported in peanut seed coat [51]. Studies have also reported the presence of these phenolic acids in peanut by-products, hull, and skin [52,53,54]. However, it was not known if they had an inhibitory effect on *A. flavus*. The radial growth bioassay performed in the present study demonstrated that both peanut soluble and insoluble biochemical seed coat extracts possess antifungal activity against *A. flavus*. The insoluble seed coat extract showed higher inhibition of *A. flavus* growth compared to soluble extracts. The HPLC data showed that the derivatives of hydroxybenzoic and hydroxycinnamic acids were the main biochemicals in the peanut seed coat (Figure 3B). Quantification of phenolics showed that vanillic acid was the most abundant phenolic acid in 55-437 and TMV-2 peanut seed coat. These results showed that ferulic acid had the highest inhibitory effect on *A. flavus*, and interestingly, ferulic acid was found to inhibit aflatoxin B_1_ production by 50% [55], suggesting the significance of ferulic acid for reduction of *A. flavus* infection and aflatoxin contamination. Further, hydroxycinnamic derivatives showed antifungal activities against *A. flavus* [25]. This suggests that the relative abundance and type of phenolic acid present in peanut seed coats differs with different varieties and could be a contributing factor in determining resistance and susceptibility among peanut genotypes. Other phenolics have been reported to have antifungal activities against different fungal species. Naturally occurring phenols, such as *p*-coumaric acids and ferulic acids, inhibited the growth of *Fusarium ananatum* in pineapples [56]. Vanillic acids and caffeic acids have been reported to inhibit the growth of fumonisin producing *Fusarium verticollides* [57], and benzoic derivatives from carnation showed antifungal activity against *Fusarium oxysporium* [58]. 

In addition to phenolic acids, the presence of other biochemicals have been reported in peanut seed coat. The presence of proanthocyanidins (tannin) as a bioactive phenolic compound has been reported in the peanut seed coat [59]. Tannin are reported to possess antifungal properties against *A. parasiticus* and reduce aflatoxin production [60]. Though there is no significant difference in the overall lignin percentage between TMV-2 and 55-437, there was a significant difference between the lignin monomer units, particularly H-lignin monomer. Hydroxycinnamic acid derivatives influence the lignin monomer composition [61] and abundance of *p*-coumaric acid influence the amount of H- monomer by increasing its monomer units deposited in the cell wall. Peanut seed coat possessed higher H-lignin and soluble proanthocyanidins in 55-437 compared to TMV-2, which correlated with higher expression of the phenylpropanoid pathway genes. The flavonoid biosynthetic genes *CHS* and *CHI* have been reported to influence the increased in pigment accumulation during peanut testa development [62]. Another study using variegated testa genotypes reported that 27 genes (*PALs*, *C4H*, *CHSs*, *F3H*, *F3’H*, *DFRs*, *LARs*, *IAAs*, *bHLHs*, and *MYBs*) involved in the pigment biosynthesis were differentially expressed between the pigmented and non-pigmented areas [63], which is consistent with the differential expression of proanthocyanidin biosynthetic genes observed between TMV-2 and 55-437. In addition to the flavonoid biosynthetic genes, transcriptional activators *AhMYB1*, *AhMYB2* and *AhTT8,* which are involved in skin-specific accumulation of anthocyanin, potentially play a role in flavanols deposition in the peanut seed coat [64]. The autoinhibited H (+)-ATPase Isoform 10 (*AHA10* or *TT13*) gene is not directly related to proanthocyanin (PA) synthesis; however, the gene plays a vital role in preparing the cellular vacuoles for uptake of PAs. Peanut *AHA10* had three times higher expression in 55-437 compared to TMV-2, indicating that the proanthocyanidins are deposited in the cellular vacuoles, which could be acting as a secondary barrier, thereby fortifying the seed coat to be resistant to *A. flavus* infection. Pathogenesis/defense-related genes showed differential expression in the seed coat of J11 when infected with *A. flavus* [65], indicating an important role of the seed coat in *A. flavus* resistance. 

Overall, the present investigation establishes that the seed coat acts as a physical and biochemical barrier against *A. flavus* infection. The role of the seed coat in protecting the cotyledons from biotic and abiotic stresses has been established in model and crop plants. A role for peanut seed coat in the *A. flavus* infection has been reported; however, there has been no systematic study on the role of seed-coat-mediated physical and biochemical resistance. Research efforts should be directed towards the developmental biology, biochemistry, and gene expression of the peanut seed coat to completely understand and utilize seed-coat-mediated *A. flavus* resistance and reduction of aflatoxin contamination. Hence, comprehensive understanding of the seed coat is essential to engineer the seed coat for improved strength and or enhanced biochemical contents for *A. flavus* resistance and aflatoxin reduction. The present study is a first step in this direction, and with the use of genomic, genetic, and biochemical studies and employment of germplasm collections, it is possible to develop and deploy seed-coat-mediated resistance in peanut. 

## Figures and Tables

**Table 1:** List of primers used for detecting toxigenic and atoxigenic *A. flavus*.

Primer Name	Sequence	Gene Bank Accession Number
*Aspergillus_ITS_F*	CGGAAGGATCATTACCGAGT	AF138287.1
*Aspergillus_ITS_R*	CCTACCTGATCCGAGGTCAA	
*aflR_F*	CTCAACGCCTCATGCTCATA	NW_002477243.1
*aflR_R*	AGCTGCCACTGTTGGTTTCT	
*aflE_norA_F*	GGAGGAAGTGATGCGAAGTC	NW_002477243.1
*aflE_norA_R*	GCTTGGGCACTGTTTCTAGC	
*omtA_afLP_F*	AGTTGGAATGTGCCTTCACC	NW_002477243.1
*omtA_afLP_R*	GCGAATTCCACTCCTTGGTA	
*aflM_F*	CCGACAACCACCGTTTAGAT	NW_002477243.1
*aflM_R*	GCTGACCTCGTCTACCTGCT	
*aflD_nor-1_F*	GGACGAGGTCTCATTGAAGC	NW_002477243.1
*aflD_nor-1_R*	TGATCATACCCGAGCACAGA	

**Table 2:** Detection and quantification data of external standard.

Phenolic Acid	Retention Time (min)	Calibration Curve Equation	R^2^	LOD (µg/mL)	LOQ (µg/mL)
Gallic acid	10.36	y = 62.359x + 725.89	0.9947	2.95	9.74
Protocatechuic acid	14.56	y = 46.285x + 338.9	0.9986	8.88	26.92
4-Hydrobenezoic acid	20.6	y = 3.4162x + 2.2449	0.9997	3.74	11.33
Caffeic acid	24.9	y = 23.06x − 53	0.9999	2.31	7.62
Vanillic acid	25.8	y = 7.4772x + 128.03	0.9961	8.63	26.15
Syringic acid	32	y = 7.8687x − 42.577	0.9973	2.5	8.25
*p*-Coumaric acid	38	y = 11.729x + 27.184	0.9962	4.5	14.85
Trans-O-Coumaric acid	44	y = 13.343x + 105.99	0.9979	3.4	11.22
Ferulic acid	47.56	y = 3.9533x + 193.77	0.9814	2.8	9.24
Sinapinic acid	53.6	y = 36.273 − 102.79	0.9999	2.07	6.29

y, peak area of the external standard; x, concentration of the external standard.

**Table 3:** List of primers used for gene expression analysis of lignin- and tannin-related genes in peanut seed coat.

Primer Name	Sequence	Gene Bank Accession Number
*CHS_F*	TCGACTCGCGAAGGATCTTG	XM_016115652.1
*CHS_R*	AACGGACGTTCCACTTTGGT	
*CHI_F*	TTCGTCAAGTTCACCGCCAT	XM_016086828.1
*CHI_R*	CGGGGTCTTACCGTTCCATT	
*F3H_F*	CCACATTCCAAAATCCGGCA	XM_016325239.1
*F3H_R*	CATCTCGGCGAAGGTGATCG	
*DFR_F*	TGCCACCAAGCCTTATCACT	XM_016117200.1
*DFR_R*	TGAATGGTGGCTTCATGTGC	
*LAR_F*	ACACTAGCTGAGAAGGCTGC	XM_016329088.1
*LAR_R*	TCTGGGGTGAGAGAAGGACC	
*AHA10_F*	AGCCATCCCCTACACCTGAT	XM_016350336.1
*AHA10_R*	AGCCATGAGTCCTTGCAGAC	
*CAD_F*	ATTGGGGCTTGGTGGAGTTG	XM_016086511.1
*CAD_R*	GGTGTCCAACAGGGACAGTG	

**Table 4:** Biochemical characterization of non-aflatoxigenic and aflatoxigenic strains from the eight *A. flavus* isolates.

Isolate	Fluorescence under UV				Remarks
	PDA Media		YES Media		
	Without B-Cyclodextrin	With B-Cyclodextrin	Without B-Cyclodextrin	With B-Cyclodextrin	
*A. flavus* -1	-	++	+	++	Toxigenic
*A. flavus* -2	-	-	+	++	Toxigenic
*A. flavus* -3	-	±	+	++	Toxigenic
*A. flavus* -4	-	-	+	++	Toxigenic
*A. flavus* -5	-	-	-	-	Atoxigenic
*A. flavus* -6	-	-	++	++	Toxigenic
*A. flavus* -7	-	-	++	++	Toxigenic
*A. flavus* -8	-	-	-	++	Toxigenic

(++), clear fluorescence; (+), weak fluorescence; (±), fluorescence not clear; (-), no fluorescence.

**Table 5:** Molecular characterization of aflatoxigenic and non-aflatoxigenic strains from the eight *A. flavus* isolates.

Isolate	Aflatoxin Producing Genes					
	*aflE*	*aflD*	*aflQ*	*aflR*	*aflM*	*omt*
*A. flavus* -1	+	+	+	+	+	+
*A. flavus* -2	+	+	+	+	+	+
*A. flavus* -3	+	+	+	+	+	+
*A. flavus* -4	+	-	+	+	+	+
*A. flavus* -5	-	-	-	+	-	-
*A. flavus* -6	+	+	+	+	-	+
*A. flavus* -7	+	-	-	-	+	-
*A. flavus* -8	+	-	-	+	+	+

(+), present; (-), absent.

## Data Availability

Not applicable.

## Supplementary Materials

The following are available online at https://www.mdpi.com/article/10.3390/jof7121000/s1, Figure S1: Visual screening of *A. flavus* isolates.
