# Supplementary material for: Peanut Seed Coat Acts as a Physical and Biochemical Barrier against Aspergillus flavus Infection
Source: J Fungi (Basel). 2021 Nov 23;7(12):1000. doi: 10.3390/jof7121000 (PMC8708384; doi:10.3390/jof7121000)
Supplement: Supplementary file 1 [file jof-07-01000-s001.zip › jof-1435713-supplementary.pdf]

A flavus isolate 1 (normal view)

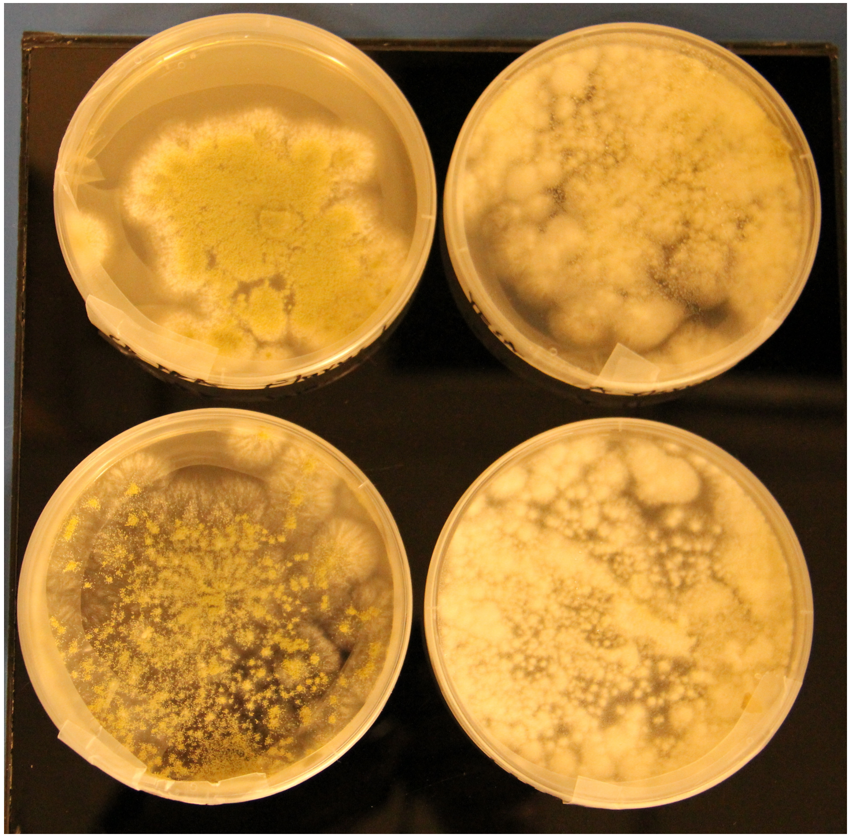

A. flavus isolate 1 (under UV)

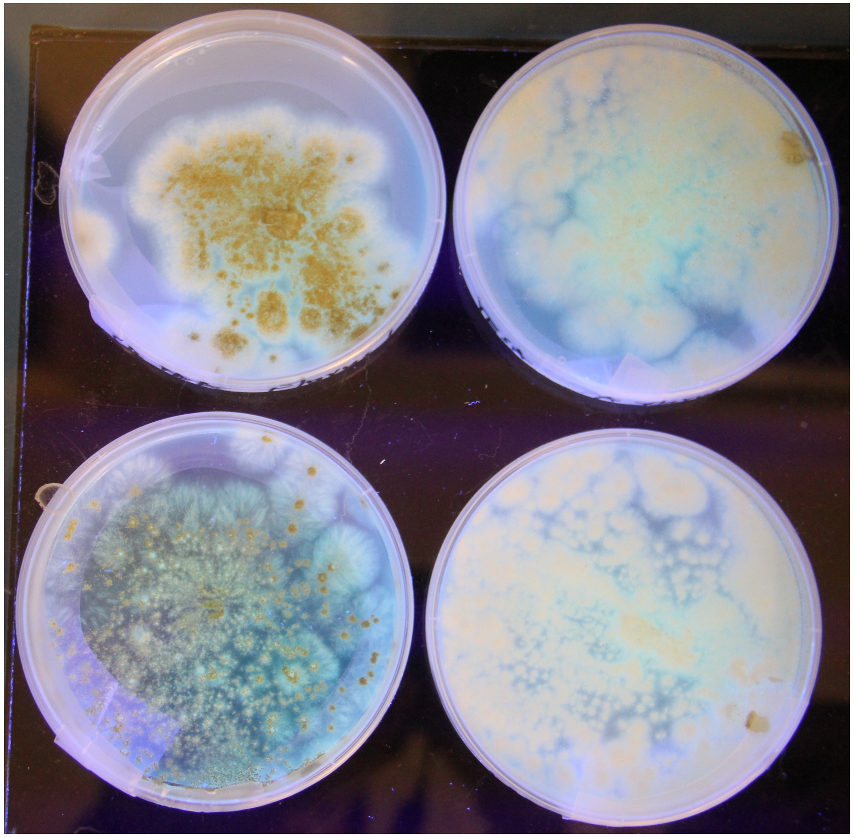

From the Top left; PDA without cyclodextrin, top right; PDA with cyclodextrin, bottom left; Yeast extract sucrose agar (YESA) without cyclodextrin, bottom right; YESA amended with cyclodextrin

Supplementary figure S1: Visual screening of *A. flavus* isolates

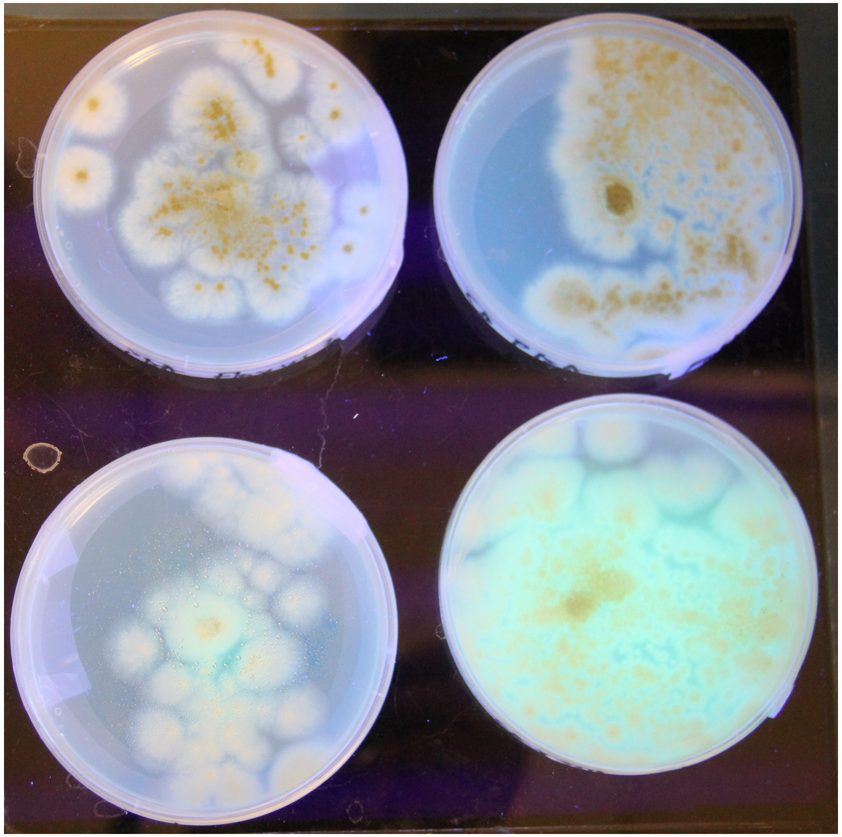

A. flavus isolate 2 (under UV)

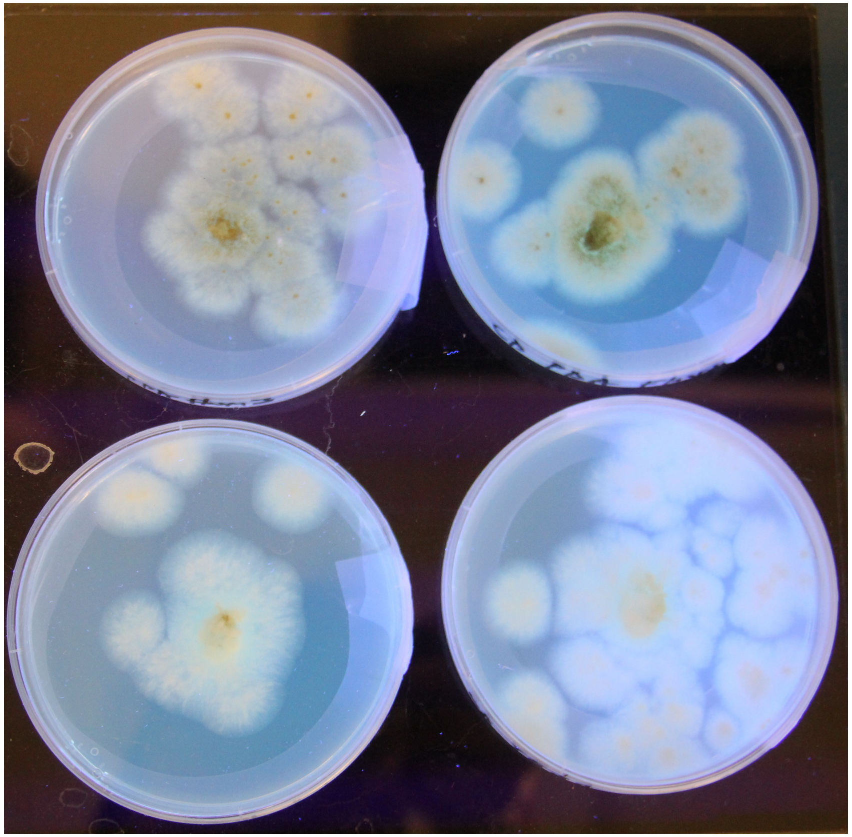

A. flavus isolate 3 (under UV)

Supplementary figures

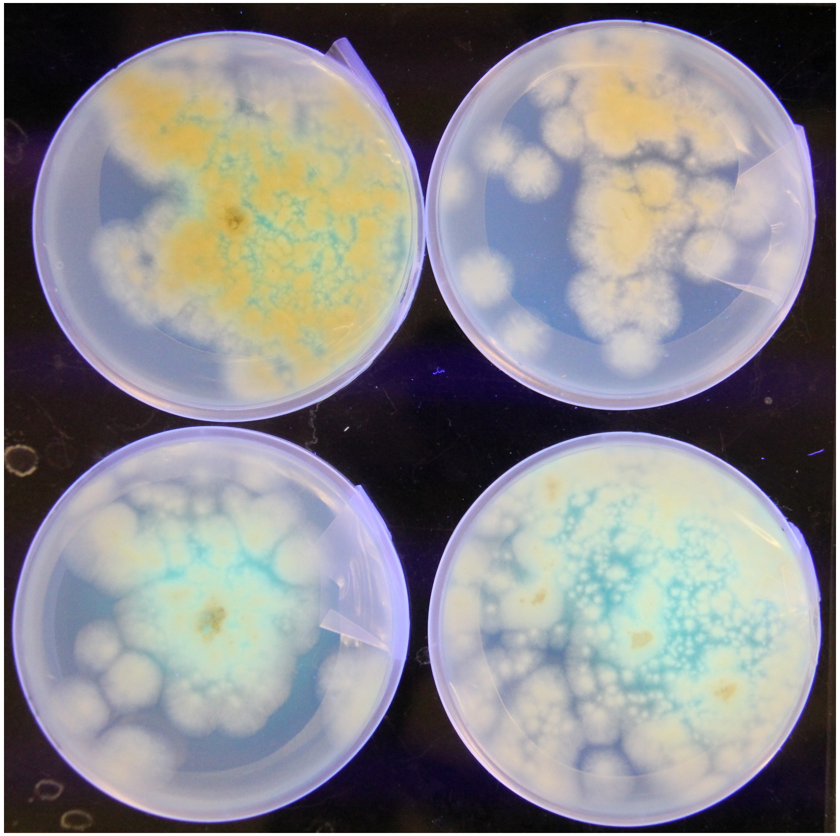

A. flavus isolate (4 under UV)

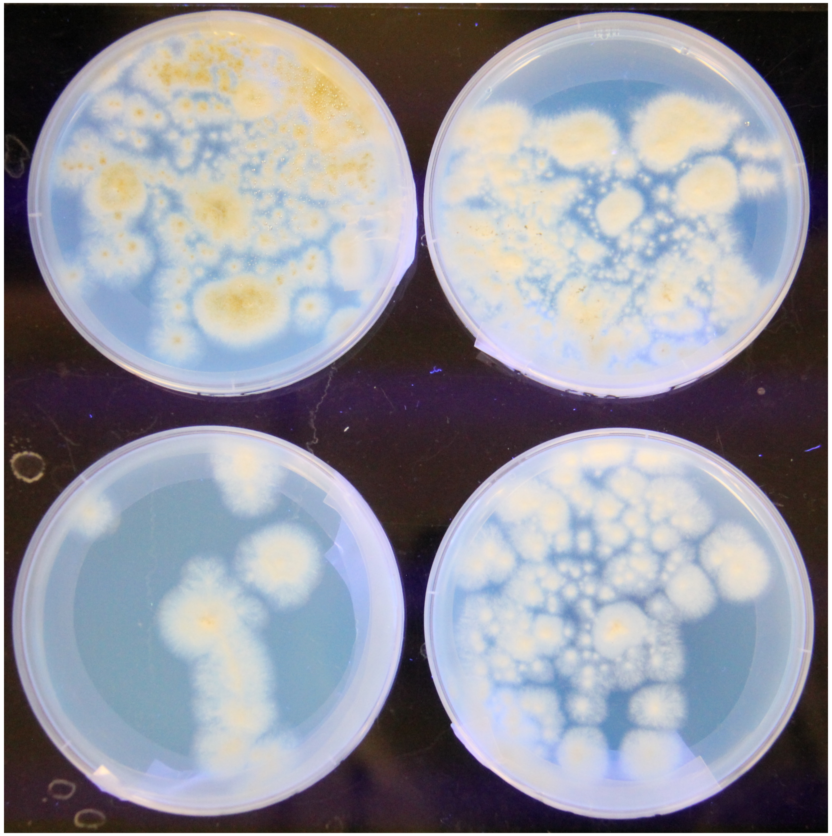

A. flavus isolate 5 (under UV)

Supplementary figures

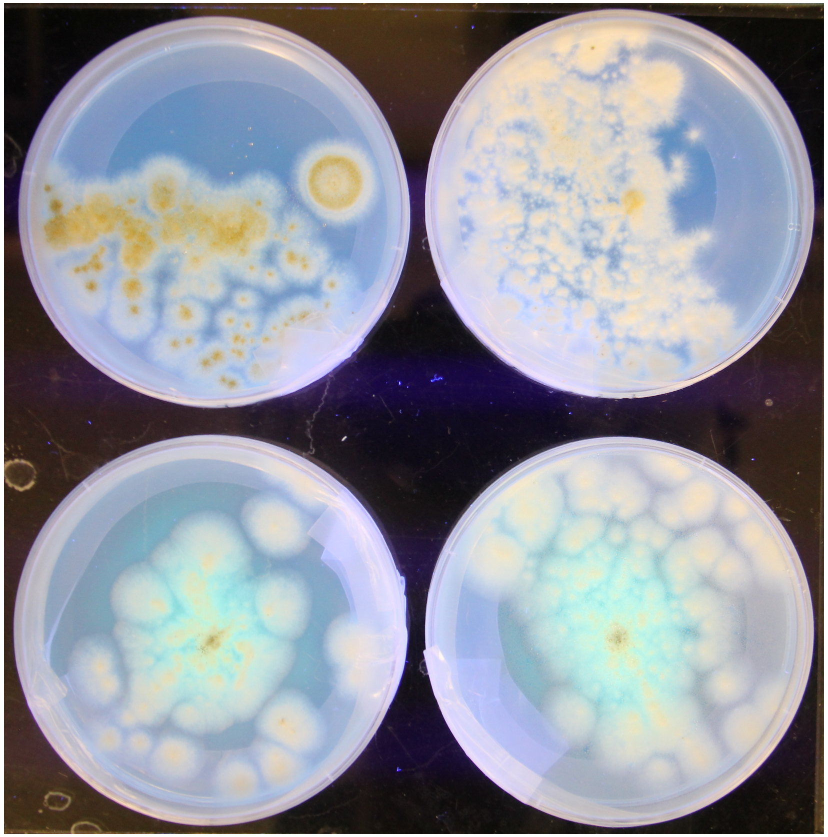

A. flavus isolate 6 (under UV)

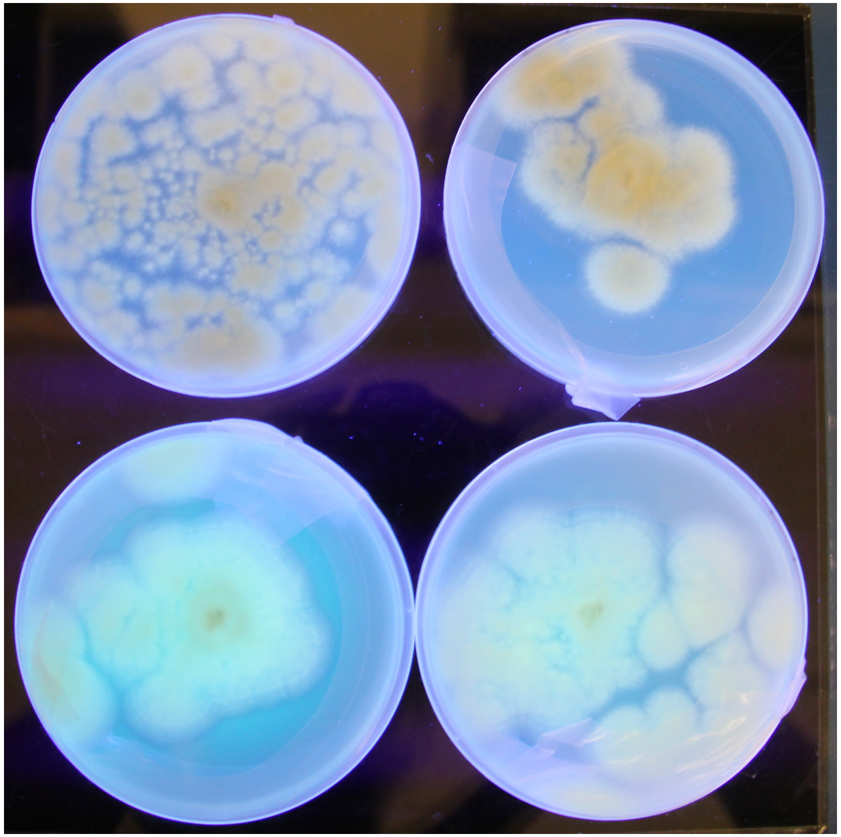

A. flavus isolate 7 (under UV)

Supplementary figures

A. flavus isolate 8 (under UV)

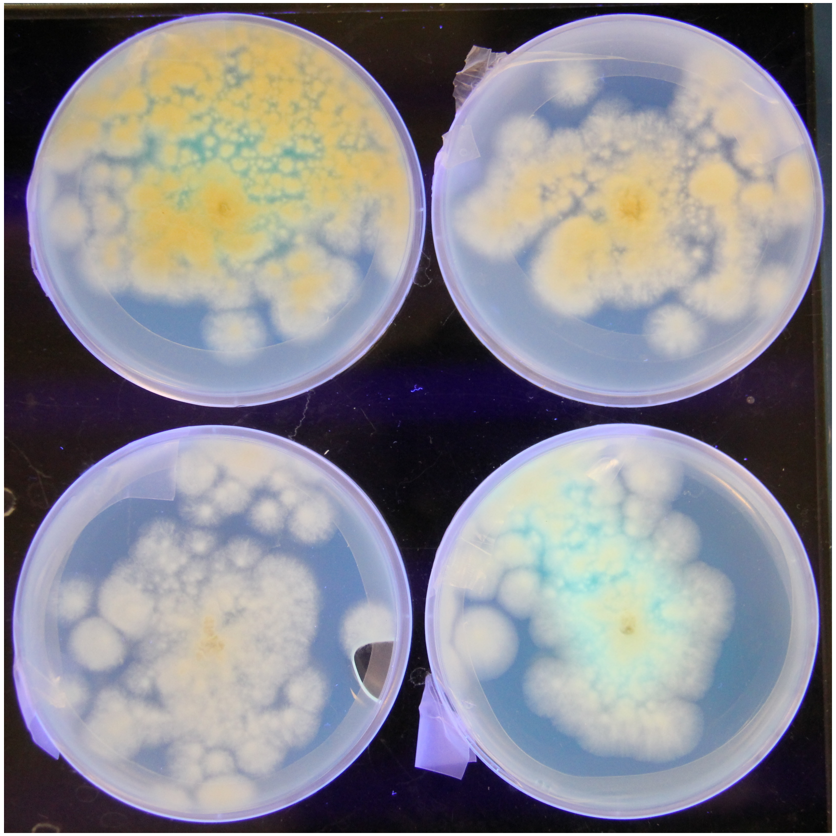

Supplementary figures
